# Accumulation and Time-Dependent Changes in Unfavorable Eating Behaviors and the Risk of Incident Metabolic Dysfunction-Associated and Alcohol-Related/Associated Steatotic Liver Disease: A Retrospective Cohort Study

**DOI:** 10.3390/nu18182957

**Published:** 2026-09-09

**Authors:** Tatsuya Fukuda, Takahiro Okamoto, Takahiro Fukaishi, Akio Kawakami, Makoto Tanaka, Tetsuya Yamada, Koshiro Monzen

**Affiliations:** 1 Mirraza Shinjuku Tsurukame Clinic, Tokyo 160-0022, Japan; 2 Department of Endocrinology and Metabolism, Tokyo Metropolitan Okubo Hospital, Tokyo 160-8488, Japan; 3 Department of Molecular Endocrinology and Metabolism, Graduate School of Medical and Dental Sciences, Institute of Science Tokyo, Tokyo 113-8510, Japan; 4 Shinjuku Tsurukame Clinic, Tokyo 151-0053, Japan; 5 Koganei Tsurukame Clinic, Tokyo 184-0004, Japan

**Keywords:** metabolic dysfunction-associated steatotic liver disease, metabolic dysfunction- and alcohol-associated liver disease, eating behavior, breakfast skipping, late-night eating, fast eating, cohort study, body mass index

## Abstract

**Background/Objectives:** Unfavorable eating behaviors may be associated with metabolic dysfunction-associated steatotic liver disease (MASLD) or metabolic dysfunction- and alcohol-associated liver disease (MetALD), but longitudinal evidence considering the accumulation and changes in these behaviors remains limited. We investigated the associations of skipping breakfast, eating dinner within 2 h before bedtime, and fast eating, individually and cumulatively, with incident MASLD/MetALD in Japanese adults undergoing repeated health examinations. **Methods:** This retrospective longitudinal cohort study included 29,889 adults without MASLD/MetALD at baseline who underwent health examinations between May 2015 and March 2026. The endpoint was defined as the development of MASLD or MetALD. An unfavorable eating behavior score ranging from 0 to 3 was calculated from the three behaviors. Cox proportional hazards models were used to estimate hazard ratios (HRs) and 95% confidence intervals (CIs). Additional analyses incorporated time-dependent eating behaviors and covariates, landmark analyses, and subgroup analyses by sex and body mass index (BMI). **Results:** During 115,088 person-years of follow-up, 4829 participants reached the endpoint. In the mutually adjusted model, skipping breakfast, eating dinner within 2 h before bedtime, and fast eating were associated with incident combined MASLD/MetALD, with HRs of 1.19 (95% CI 1.11–1.27), 1.11 (1.05–1.18), and 1.06 (1.00–1.13), respectively. Compared with participants reporting no unfavorable eating behaviors, the fully adjusted HRs were 1.12 (1.04–1.20), 1.22 (1.13–1.33), and 1.44 (1.28–1.61) for those reporting 1, 2, and 3 behaviors, respectively. Each additional behavior was associated with an HR of 1.12 (1.08–1.15). The association of the cumulative score remained evident in time-dependent and landmark analyses. The cumulative-score association was stronger among participants with BMI < 23 kg/m^2^ than among those with BMI ≥ 23 kg/m^2^ (*p* for interaction < 0.001), whereas no statistically significant interaction by sex was observed. **Conclusions:** The accumulation of unfavorable eating behaviors was associated with a progressively higher incidence of MASLD/MetALD. Repeated assessment of simple eating behaviors during routine health examinations may help identify individuals at elevated risk, including those below conventional obesity thresholds.

## 1. Introduction

Metabolic dysfunction-associated steatotic liver disease (MASLD) is characterized by hepatic steatosis in the presence of at least one cardiometabolic risk factor. Under the new steatotic liver disease nomenclature, individuals who have the same metabolic phenotype but consume moderate amounts of alcohol are classified as having metabolic dysfunction- and alcohol-associated liver disease (MetALD) [1]. Because MASLD and MetALD share hepatic steatosis and cardiometabolic dysfunction as core diagnostic features and are differentiated within the consensus framework according to the amount of alcohol consumed, the present study evaluated incident MASLD or MetALD as a prespecified combined endpoint. Together, these conditions represent a substantial burden of chronic liver disease worldwide and are associated with progressive liver disease, type 2 diabetes, cardiovascular disease, and excess mortality [2,3]. Identifying modifiable exposures that can be assessed before disease is therefore an important public health priority.

Nutrition and lifestyle modification are central to the prevention and management of MASLD [2]. Studies evaluating overall dietary quality have generally reported lower odds of MASLD or non-alcoholic fatty liver disease among individuals with greater adherence to healthy eating indices, Dietary Approaches to Stop Hypertension, or Mediterranean-style patterns, whereas greater consumption of ultra-processed foods has been associated with higher odds of disease [4,5,6,7,8]. These findings support a pattern-based approach to nutrition; however, detailed dietary assessment is time-consuming and may be difficult to repeat during routine health examinations.

The timing and manner of food intake may provide a complementary and highly actionable dimension of nutritional assessment. Chrononutrition research suggests that irregular or late eating can misalign feeding-related signals with endogenous circadian rhythms and adversely affect glucose tolerance, insulin sensitivity, lipid handling, appetite regulation, and energy balance [9,10,11,12,13,14,15,16]. Because feeding is a major synchronizer of peripheral clocks, including the hepatic clock, habitual meal timing may be relevant to the development of hepatic steatosis even when total dietary intake is not measured in detail [10,11,12,14,15].

Several commonly assessed eating behaviors may capture this dimension. A Japanese retrospective cohort linked eating within 2 h before bedtime, particularly in combination with fast eating, to incident non-alcoholic fatty liver disease [17]. More recently, Takahata et al. evaluated several lifestyle and eating behaviors, including fast eating, eating before bedtime, and skipping breakfast, in relation to incident non-alcoholic fatty liver disease in a longitudinal cohort of Japanese adults [18]. Fast eating has also been associated with non-alcoholic fatty liver disease in men with type 2 diabetes and with obesity in meta-analyses [19,20], while breakfast skipping has been associated with a higher risk of type 2 diabetes in prospective studies and has been examined in relation to fatty liver in population-based data [21,22]. Thus, longitudinal evidence has linked individual eating behaviors and selected combinations of behaviors to incident fatty liver disease. However, evidence remains limited regarding whether the cumulative burden of multiple unfavorable eating behaviors is associated with incident MASLD/MetALD. In addition, few studies have incorporated repeated or time-updated assessments of eating behaviors during follow-up to account for changes in these behaviors over time.

This evidence gap may be especially relevant in East Asian populations, in which a substantial proportion of steatotic liver disease occurs below conventional obesity thresholds [23,24,25]. In this setting, behavioral information not captured by BMI alone may help identify individuals at risk. Prospective evidence is particularly limited regarding the cumulative burden of multiple unfavorable eating behaviors, repeated or time-updated assessment of these behaviors during follow-up, and possible heterogeneity according to BMI.

Therefore, the present study investigated the longitudinal associations of skipping breakfast, eating dinner within 2 h before bedtime, and fast eating with incident MASLD/MetALD in a large cohort of Japanese adults undergoing repeated health examinations. We hypothesized that the accumulation of these behaviors would be associated with progressively higher MASLD/MetALD incidence. We also examined time-updated exposures and covariates, evaluated associations according to sex and BMI category, and performed landmark analyses to assess the robustness of the findings.

## 2. Materials and Methods

### 2.1. Study Design and Data Source

This was a retrospective longitudinal cohort study using data from a health examination database. The study included individuals who underwent health examinations at the Mirraza Shinjuku Tsurukame Clinic, Koganei Tsurukame Clinic, and Shinjuku Tsurukame Clinic (Tokyo, Japan) between 1 May 2015 and 31 March 2026. The data source consisted of routinely collected health examination records, including questionnaire-based lifestyle information, anthropometric measurements, laboratory data, medication and medical history information, and abdominal ultrasonography findings.

The unit of analysis was the individual participant rather than the individual health examination record. For each participant, the first available health examination during the study period was defined as the index examination and served as baseline. Baseline information was used to determine eligibility, define baseline eating behaviors and covariates, and assess the presence or absence of MASLD/MetALD at study entry. Subsequent health examinations were used to ascertain incident MASLD/MetALD during follow-up.

Participants were followed from the date of the baseline examination until the first detection of MASLD/MetALD or the last available health examination, whichever came first. For the primary analysis, exclusion criteria were determined using baseline information, whereas post-baseline information was not used to determine initial eligibility.

The study protocol was approved by the ethics committee of Shinjuku Tsurukame Clinic (approval no. 2603). Because this was a retrospective study using existing health examination data, the requirement for written informed consent was waived, and information on the study was disclosed in accordance with the opt-out policy of the institution. The study was conducted in accordance with the principles of the Declaration of Helsinki.

### 2.2. Participants

Participants were eligible if they underwent a baseline health examination during the study period and had sufficient baseline information to determine the presence or absence of MASLD/MetALD. Individuals were excluded if they were aged <20 years (*n* = 21), had excessive alcohol consumption (*n* = 1259), had a history of malignancy (*n* = 1545), had thyroid disease (*n* = 31), had viral hepatitis (*n* = 1556), or had missing baseline data required for defining eligibility, exposures, outcomes, or covariates (*n* = 918) at baseline (Supplementary Figure S1). Among eligible individuals, those with MASLD/MetALD at baseline were excluded. Participants without MASLD/MetALD at baseline were followed from the baseline examination until the first detection of MASLD/MetALD or the last available health examination, whichever came first. Individuals without any follow-up health examination after baseline were not included in the incidence cohort. The final incidence cohort comprised 29,889 individuals without MASLD/MetALD at baseline who underwent at least one subsequent health examination.

### 2.3. Assessment of Eating Behaviors

Eating behaviors were assessed using standardized self-administered questionnaires completed at each health examination. The exposures of interest were skipping breakfast, eating dinner within 2 h before bedtime, and fast eating. Skipping breakfast was defined as not eating breakfast at least three times per week. Eating dinner within 2 h before bedtime was defined as eating within 2 h before sleep at least three times per week. Fast eating was defined as self-reported eating at a faster speed than others. Each behavior was coded as a binary variable, with 0 indicating the absence and 1 indicating the presence of the corresponding behavior.

An unfavorable eating behavior score was calculated as the sum of skipping breakfast, eating dinner within 2 h before bedtime, and fast eating, with one point assigned for the presence of each behavior. The score therefore ranged from 0 to 3. For the baseline analyses, the score was calculated using responses obtained at the baseline health examination. For the time-dependent analyses, the score was recalculated at the beginning of each observation interval using responses obtained at the corresponding health examination.

### 2.4. Ultrasonographic Assessment and Definition of Incident MASLD or MetALD

Abdominal ultrasonography was performed by trained clinical laboratory technologists using the Aplio MX, Aplio 300, Aplio a450, or Aplio a550 ultrasound systems (Canon Medical Systems, Tokyo, Japan). Hepatic steatosis was diagnosed when at least one of the following findings was present: increased hepatic echogenicity (bright liver), hepatorenal contrast, obscuration of intrahepatic vessels, or deep attenuation. The initial ultrasonographic assessment was performed by a clinical laboratory technologist, and the final diagnosis was confirmed by a board-certified gastroenterologist after review of the ultrasound images. The questionnaire definitions and ultrasonographic assessment procedures were consistent with those used in our previous health-checkup study [26].

According to the multisociety consensus statement [1], conventional MASLD and MetALD are both defined by hepatic steatosis and at least one cardiometabolic risk factor, with classification according to the amount of alcohol consumed. Accordingly, the primary outcome was defined as incident MASLD or MetALD, hereafter referred to as the combined MASLD/MetALD outcome. This combined outcome was used to capture metabolically associated steatotic liver disease across the alcohol-consumption ranges encompassed by the two diagnostic categories, while excluding alcohol consumption above the MetALD range. Participants were classified as having the combined MASLD/MetALD outcome if they had hepatic steatosis on abdominal ultrasonography, at least one of the five cardiometabolic risk factors described below, and weekly alcohol consumption of ≤350 g in women or ≤420 g in men. Conventional MASLD was defined by alcohol consumption of <140 g/week in women or <210 g/week in men, whereas MetALD was defined by alcohol consumption of 140–350 g/week in women or 210–420 g/week in men. Participants consuming >350 g/week in women or >420 g/week in men were excluded.

The five cardiometabolic risk factors were: (1) BMI ≥ 23 kg/m^2^ or waist circumference ≥ 85 cm in men or ≥90 cm in women; (2) fasting plasma glucose ≥ 100 mg/dL or treatment for type 2 diabetes; (3) blood pressure ≥ 130/85 mmHg or antihypertensive treatment; (4) triglycerides ≥ 150 mg/dL or lipid-lowering treatment; and (5) HDL cholesterol ≤ 40 mg/dL in men or ≤50 mg/dL in women or lipid-lowering treatment.

The primary outcome was the combined MASLD/MetALD outcome during follow-up. Among individuals without combined MASLD/MetALD at baseline, incident combined MASLD/MetALD was defined as the first subsequent health examination at which this combined outcome was identified. For individuals who developed the combined MASLD/MetALD outcome, follow-up ended on the date of the first examination showing combined MASLD/MetALD. Individuals who did not develop the combined outcome were censored on the date of their last examination confirming the absence of combined MASLD/MetALD.

### 2.5. Covariates

The covariates considered in the analyses were age, sex, smoking status, log-transformed alcohol consumption, regular exercise, physical activity, self-reported adequate rest from sleep, body mass index (BMI), fasting plasma glucose (FPG), systolic blood pressure (SBP), triglycerides (TG), and high-density lipoprotein cholesterol (HDL-C). Smoking status was categorized as current or non-current smoking. Regular exercise was defined as exercise for at least 30 min per session, at least twice per week, for at least 1 year. Physical activity was defined as walking or an equivalent activity for at least 1 h/day. Adequate rest from sleep was defined as waking feeling well rested.

Weekly alcohol consumption was estimated from self-reported drinking frequency and the amount consumed per drinking occasion. One drink was standardized as approximately 20 g of alcohol. Reported amounts of <1, 1–2, 2–3, and ≥3 drinks per occasion were assigned 20, 30, 50, and 70 g, respectively, and multiplied by 7 for daily drinkers and by 2 for occasional drinkers; rare drinkers were assigned 0 g/week.

In the baseline analyses, all eating behaviors and covariates were fixed at their baseline values. In the exploratory analyses with time-dependent covariates, age, smoking status, alcohol consumption, exercise, physical activity, sleep, BMI, FPG, SBP, TG, and HDL-C were updated using the values recorded at the beginning of each observation interval, whereas sex was treated as a fixed covariate.

### 2.6. Statistical Analysis

Continuous variables were summarized as means and standard deviations or medians and interquartile ranges, as appropriate, whereas categorical variables were presented as numbers and percentages. Baseline characteristics were compared across groups defined by the number of unfavorable eating behaviors. Welch’s one-way analysis of variance was used for approximately normally distributed continuous variables, the Kruskal–Wallis rank-sum test was used for skewed continuous variables, and Pearson’s chi-squared test was used for categorical variables.

The incidence rate of MASLD/MetALD was calculated by dividing the number of incident cases by the total person-years of observation and was expressed per 1000 person-years. Associations between individual eating behaviors and incident MASLD/MetALD were initially evaluated using Cox proportional hazards regression models in which eating behaviors and covariates were fixed at their baseline values. Hazard ratios (HRs) and 95% confidence intervals (CIs) were estimated, and tied event times were handled using the Efron method.

The crude model included each eating behavior alone. Model 1 was adjusted for age, sex, smoking status, and log-transformed alcohol consumption. Model 2 was additionally adjusted for regular exercise, physical activity, and adequate rest from sleep. Model 3 was further adjusted for BMI, and Model 4 was additionally adjusted for FPG, SBP, TG, and HDL-C.

Each eating behavior was first evaluated separately. Skipping breakfast, eating dinner within 2 h before bedtime, and fast eating were subsequently entered simultaneously into Model 4 to assess whether each behavior was associated with incident MASLD/MetALD independently of the other two eating behaviors.

Associations between the number of unfavorable eating behaviors at baseline and incident MASLD/MetALD were evaluated using the same sequential Cox proportional hazards models. The eating behavior score was analyzed categorically, with a score of 0 used as the reference category, and continuously to estimate the HR associated with each additional unfavorable eating behavior. Incidence rates were calculated separately for participants with 0, 1, 2, and 3 unfavorable eating behaviors.

Exploratory subgroup analyses were conducted to assess whether the association between the unfavorable eating behavior score and incident MASLD/MetALD differed according to sex or baseline BMI category (<23 or ≥23 kg/m^2^). In these analyses, the eating behavior score was modeled continuously to estimate the HR associated with each additional unfavorable eating behavior. Sex-stratified models were adjusted for all Model 4 covariates except sex, whereas BMI-stratified models were adjusted for all Model 4 covariates except BMI. Effect modification was evaluated in the overall cohort by including a multiplicative interaction term between the unfavorable eating behavior score and the corresponding effect modifier. *p*-values for interaction were obtained using likelihood ratio tests comparing nested Cox models with and without the interaction term.

The proportional hazards assumption was assessed using Schoenfeld residuals. Because evidence of nonproportionality was detected for sex and several continuous covariates, extended Cox models were fitted as sensitivity analyses. In these models, the baseline hazard was stratified by sex, and time-varying coefficients were specified for age, FPG, SBP, and TG. These continuous variables were centered at their respective means, and their time-varying effects were modeled by including interactions with log(t + 0.01), where t represented follow-up time in years. These sensitivity analyses were performed for both the mutually adjusted individual eating behavior model and the unfavorable eating behavior score model.

To account for changes in eating behaviors during follow-up, additional Cox proportional hazards models with time-dependent covariates were fitted using a start–stop, or counting-process, data structure. Each participant’s follow-up period was divided into intervals between consecutive health examinations. The eating behaviors recorded at the beginning of each interval were used as exposures for the period extending to the subsequent examination. When MASLD/MetALD was first identified at the subsequent examination, an event was assigned to the end of that interval. Observation intervals occurring after the first MASLD/MetALD-positive examination were excluded.

In the first analysis with time-dependent individual eating behaviors, skipping breakfast, eating dinner within 2 h before bedtime, and fast eating were updated at each health examination, whereas the adjustment covariates were fixed at their baseline values. The three eating behaviors were simultaneously included in the fully adjusted model.

In the corresponding analysis of the time-dependent eating behavior score, the score was recalculated at the beginning of each observation interval, whereas adjustment covariates were fixed at their baseline values. The score was analyzed both categorically, with 0 unfavorable eating behaviors used as the reference category, and continuously per one-behavior increment.

In further exploratory analyses, both the eating behaviors and the available demographic, lifestyle, anthropometric, and metabolic covariates were updated using values recorded at the beginning of each observation interval. Separate models were fitted for the three individual eating behaviors and for the unfavorable eating behavior score.

Because each individual could contribute multiple observation intervals, robust variance estimates clustered by participant identification number were used in all time-dependent Cox models to account for within-participant correlation.

To assess the potential influence of reverse causation due to subclinical MASLD/MetALD present shortly after baseline, 1-year and 2-year landmark sensitivity analyses were performed. Participants who remained free of MASLD/MetALD and under observation beyond the corresponding landmark time were included, and follow-up time was recalculated from the landmark time. Participants who developed MASLD/MetALD or were censored on or before the landmark time were excluded. Baseline eating behaviors and covariates were retained in these analyses. The mutually adjusted model for the three individual eating behaviors and the fully adjusted models for the categorical and continuous unfavorable eating behavior score were refitted using the same covariates as Model 4.

A total of 29,889 individuals in the final incidence cohort were included in the primary analyses. The smaller sample sizes in the landmark analyses resulted from the requirement that participants remain free of MASLD/MetALD and under observation beyond the corresponding landmark time, rather than from missing data. No *a priori* sample-size calculation was performed because this was a retrospective cohort study using an existing health examination database, and all individuals who met the prespecified baseline eligibility criteria and underwent at least one follow-up health examination during the study period were included. In the final incidence cohort, 4829 incident MASLD/MetALD events occurred among 29,889 participants, and statistical precision was evaluated based on the width of the 95% confidence intervals around the hazard ratio estimates. All statistical tests were two-sided, and *p*-values < 0.05 were considered statistically significant. All analyses were performed using R version 4.6.0 (R Foundation for Statistical Computing, Vienna, Austria) and the survival package.

### 2.7. Use of Generative Artificial Intelligence

During the preparation of this manuscript, the authors used ChatGPT (GPT-5.5 Thinking; OpenAI, San Francisco, CA, USA) to assist with drafting and language refinement. The authors critically reviewed and edited all generated content, verified the cited sources and numerical results, and take full responsibility for the final content of the manuscript. Generative artificial intelligence was not used for study design, data collection, statistical analysis, or interpretation of the study results.

## 3. Results

### 3.1. Study Population and Incidence of MASLD/MetALD

The final incidence cohort comprised 29,889 individuals without MASLD/MetALD at baseline. Among the 53,603 eligible individuals without MASLD/MetALD at baseline, 29,889 underwent at least one subsequent health examination and were included in the incidence cohort. Baseline characteristics of participants included in the incidence cohort and those without a follow-up examination are shown in Supplementary Table S1.

During 115,088 person-years of follow-up, 4829 individuals developed MASLD/MetALD, whereas 25,060 remained free of MASLD/MetALD. The incidence rate of MASLD/MetALD was 42.0 per 1000 person-years. The median follow-up duration was 2.98 years, with an interquartile range of 1.06–5.81 years.

Baseline characteristics according to the number of unfavorable eating behaviors are shown in Table 1. At baseline, 10,441 participants had no unfavorable eating behaviors, 11,343 had one behavior, 6392 had two behaviors, and 1713 had all three behaviors.

### 3.2. Associations Between Individual Eating Behaviors and Incident MASLD/MetALD

Table 2 shows the associations between individual unfavorable eating behaviors and incident MASLD/MetALD. In the fully adjusted Model 4, skipping breakfast, eating dinner within 2 h before bedtime, and fast eating were each associated with incident MASLD/MetALD. When the three eating behaviors were included simultaneously in the fully adjusted model, the adjusted HRs were 1.19 (95% CI, 1.11–1.27; *p* < 0.001) for skipping breakfast, 1.11 (95% CI, 1.05–1.18; *p* = 0.001) for eating dinner within 2 h before bedtime, and 1.06 (95% CI, 1.00–1.13; *p* = 0.034) for fast eating.

### 3.3. Associations Between the Number of Unfavorable Eating Behaviors and Incident MASLD/MetALD

Incidence rates and Cox regression results according to the number of unfavorable eating behaviors are shown in Table 3. The incidence rate of MASLD/MetALD increased from 32.2 per 1000 person-years among participants with no unfavorable eating behaviors to 65.0 per 1000 person-years among those with all three behaviors. In the fully adjusted Model 4, compared with participants with no unfavorable eating behaviors, the adjusted HRs were 1.12 (95% CI, 1.04–1.20; *p* = 0.003), 1.22 (95% CI, 1.13–1.33; *p* < 0.001), and 1.44 (95% CI, 1.28–1.61; *p* < 0.001) for participants with 1, 2, and 3 unfavorable eating behaviors, respectively. Each additional unfavorable eating behavior was associated with an adjusted HR of 1.12 (95% CI, 1.08–1.15; *p* < 0.001).

In time-dependent analyses, each additional unfavorable eating behavior was associated with incident MASLD/MetALD when the eating behavior score was updated at each health examination with baseline covariates fixed (HR, 1.13; 95% CI, 1.10–1.18; *p* < 0.001) and when both the eating behavior score and covariates were updated over time (HR, 1.09; 95% CI, 1.05–1.13; *p* < 0.001).

### 3.4. Proportional Hazards Assumption and Sensitivity Analyses

The proportional hazards assumption was assessed using Schoenfeld residuals. Because evidence of nonproportionality was observed for sex and several continuous covariates, extended Cox models were fitted as sensitivity analyses. In these models, the associations of individual eating behaviors and the unfavorable eating behavior score were similar to those in the primary analyses (Supplementary Tables S2 and S3).

In landmark sensitivity analyses, each additional unfavorable eating behavior was associated with incident MASLD/MetALD in both the 1-year landmark analysis (HR, 1.12; 95% CI, 1.08–1.16; *p* < 0.001) and the 2-year landmark analysis (HR, 1.09; 95% CI, 1.04–1.13; *p* < 0.001) (Table 4). Skipping breakfast was associated with incident MASLD/MetALD in both landmark analyses, whereas the associations for eating dinner within 2 h before bedtime and fast eating were not statistically significant in the 2-year landmark analysis.

Additional time-dependent analyses of individual eating behaviors are shown in Supplementary Table S4. The model with updated eating behaviors and baseline covariates included 82,479 observation intervals contributed by 29,889 participants, with 4829 incident MASLD/MetALD events. In this model, all three individual eating behaviors were associated with incident MASLD/MetALD. In the fully updated model, which included 82,427 observation intervals and 4827 events after excluding 52 intervals with missing updated covariate values, skipping breakfast and eating dinner within 2 h before bedtime remained statistically significant, whereas fast eating was not statistically significant.

In an additional sensitivity analysis censoring participants at the first follow-up examination with newly identified exclusion criteria, the cumulative unfavorable eating behavior score remained associated with incident MASLD/MetALD, with an adjusted HR of 1.11 (95% CI, 1.07–1.15; *p* < 0.001) per 1-behavior increment (Supplementary Table S5).

### 3.5. Subgroup Analyses

Subgroup analyses are shown in Table 5. The association between the unfavorable eating behavior score and incident MASLD/MetALD was observed in both women and men, with no statistically significant interaction by sex (*p* for interaction = 0.109). The association was also observed in both BMI categories; the HR per 1-behavior increment was 1.19 (95% CI, 1.14–1.24; *p* < 0.001) among participants with BMI < 23 kg/m^2^ and 1.08 (95% CI, 1.03–1.13; *p* = 0.002) among those with BMI ≥ 23 kg/m^2^. The interaction between the eating behavior score and BMI category was statistically significant (*p* for interaction < 0.001).

## 4. Discussion

In this longitudinal cohort of 29,889 Japanese adults without MASLD/MetALD at baseline, three readily assessed eating behaviors were longitudinally associated with subsequent MASLD/MetALD in the primary analyses. Skipping breakfast showed the most consistent individual association, whereas the estimates for eating dinner within 2 h before bedtime and fast eating were smaller and were attenuated in some sensitivity analyses. In addition, the cumulative number of unfavorable eating behaviors showed a graded association with incident MASLD/MetALD. Compared with participants reporting no unfavorable behaviors, the fully adjusted hazard ratios were 1.12, 1.22, and 1.44 for one, two, and three behaviors, respectively, and each additional behavior was associated with a 12% higher hazard. The association remained evident when the score was updated at repeated examinations, when available covariates were also updated, in 1- and 2-year landmark analyses, and in the sensitivity analysis censoring participants at the first follow-up examination with newly identified exclusion criteria. Because this was an observational study, these findings should be interpreted as associations and do not establish that these eating behaviors cause MASLD/MetALD or that modifying them would necessarily prevent disease onset.

The present findings should be interpreted in the context of previous longitudinal evidence. Prior studies have reported associations of individual eating behaviors, including eating before bedtime, fast eating, and skipping breakfast, with incident non-alcoholic fatty liver disease. Thus, the present study should not present the general association between unfavorable eating behaviors and incident fatty liver disease as an entirely new finding. Rather, its main contribution is the evaluation of the cumulative burden of multiple unfavorable eating behaviors, the use of repeated/time-updated assessments to account for changes in these behaviors during follow-up, and the examination of these associations using incident MASLD/MetALD as the outcome.

The graded association of the unfavorable eating behavior score complements evidence derived from broader measures of dietary quality. Greater adherence to healthy eating indices and Mediterranean-style dietary patterns has been associated with lower odds of MASLD, whereas higher ultra-processed food intake and poorer dietary quality have been associated with higher odds [4,5,6,7,8]. Our findings extend this literature from food- and nutrient-based patterns to a compact behavior-based score obtainable from three questionnaire items. This score cannot replace a comprehensive nutritional assessment, but it may capture a cluster of behaviors related to meal regularity, circadian alignment, appetite control, and energy intake. Its association after adjustment for physical activity, sleep-related rest, BMI, glucose, blood pressure, triglycerides, and HDL cholesterol suggests that it provides information beyond conventional metabolic risk markers.

Skipping breakfast was the most robust individual behavior. It remained associated with incident MASLD/MetALD in the mutually adjusted baseline model, the time-dependent model with baseline covariates, the exploratory model with updated covariates, and both landmark analyses. Breakfast skipping may be a marker of broader lifestyle disruption; however, plausible metabolic pathways also exist. Omitting the first meal can shift a larger proportion of daily energy intake to later hours, impair appetite regulation, and increase glycemic and insulin responses to subsequent meals [9,13,14,15,16,27,28]. Prospective evidence has also linked breakfast skipping to type 2 diabetes, with partial attenuation after adjustment for BMI [21]. Consistent with that pattern, BMI adjustment only modestly reduced the estimate for breakfast skipping in our study, although residual confounding by total energy intake, diet composition, chronotype, shift work, and socioeconomic factors remains possible.

Eating dinner within 2 h before bedtime was independently associated with MASLD/MetALD in the primary model and when eating behaviors were updated, including in the fully updated model, although the estimate was attenuated and the association was not statistically significant in the 2-year landmark analysis. Previous Japanese data similarly linked eating before bedtime, particularly in combination with fast eating, to the development of non-alcoholic fatty liver disease [17]. Late eating occurs during a biological period characterized by lower glucose tolerance and may misalign feeding cues with central and hepatic circadian clocks [9,10,11,12,13,14,15,16,28]. The attenuation in the more conservative analyses suggests that part of the association may operate through subsequent changes in weight or metabolic status, may reflect unmeasured shift work or sleep patterns, or may be especially susceptible to behavioral misclassification.

Fast eating showed the smallest independent estimate and was most strongly attenuated after BMI was added to the model. Rapid eating has been associated with obesity in meta-analyses and with non-alcoholic fatty liver disease in men with type 2 diabetes [19,20]. A higher eating rate may shorten oral processing and satiety signaling, facilitate excess energy intake, and worsen postprandial glycemia [13]. The marked attenuation after BMI adjustment is compatible with adiposity acting either as an intermediate pathway or as a major confounder. Because BMI may partly lie downstream of habitual eating speed, models adjusted for baseline or updated BMI estimate an association that excludes at least part of the pathway through weight gain. The remaining modest association should therefore be interpreted cautiously, and the null result in the fully updated model should not be interpreted as excluding a possible pathway through adiposity.

Because detailed dietary intake was not assessed, the present study cannot determine whether the observed associations were driven primarily by chrononutrition-related mechanisms, unmeasured differences in total energy intake, or both [13,15,16]. This distinction may be particularly important for skipping breakfast and fast eating. Breakfast skipping may reflect delayed meal timing and circadian disruption, but it may also be associated with compensatory energy intake later in the day and metabolic risk [13,21]. Similarly, fast eating may influence postprandial metabolism, but it may also promote excess energy intake through reduced oral processing and delayed satiety signaling, thereby contributing to adiposity [13,20]. Therefore, the observed associations should be interpreted as reflecting behavioral patterns that may incorporate both meal-timing and energy-intake pathways, rather than as evidence for a purely chrononutrition-related mechanism.

The repeated-measures analyses provide complementary information because eating behaviors can change over time and reliance on baseline responses alone may produce exposure misclassification. Updating the eating behavior score at each examination yielded an association similar to that of the baseline model when covariates were fixed at baseline. When both the score and available covariates were updated, the estimates were attenuated but remained graded, with a 9% higher hazard per additional behavior. This latter model may better reflect concurrent metabolic status, but it may also overadjust for time-varying factors such as BMI, glucose, and triglycerides that could lie on the causal pathway from earlier eating behavior to MASLD. The baseline, time-updated exposure, and fully updated models should therefore be viewed as complementary rather than as progressively superior estimates. The landmark analyses further reduced concern that unrecognized steatosis shortly after baseline fully explained the findings, although reverse causation cannot be eliminated completely.

The relative association of the eating behavior score was stronger among participants with BMI < 23 kg/m^2^ than among those with BMI ≥ 23 kg/m^2^, whereas there was no statistically significant interaction by sex. The BMI finding is potentially relevant because non-obese and lean steatotic liver disease represents a substantial proportion of disease worldwide and can still be associated with adverse hepatic and metabolic outcomes [23,25]. In Japan, fatty liver is common despite lower average BMI than in many Western populations [24]. Unfavorable eating behaviors may therefore provide risk information that is not apparent from BMI alone. Nevertheless, the interaction analysis was exploratory, and a stronger relative association in the lower-BMI group does not imply a lower absolute disease burden among participants with higher BMI.

The absence of statistically significant effect modification by sex differs from a prior cross-sectional study in patients with type 2 diabetes, in which fast eating was associated with non-alcoholic fatty liver disease in men but not women [19]. The present study included a broader health-examination population, evaluated incident disease, and focused primarily on the cumulative score rather than fast eating alone. These design differences may explain the more similar associations observed between women and men. The modest estimate among women and the exploratory nature of the subgroup analyses argue against strong claims of sex-specific benefit or harm.

From a clinical perspective, the findings suggest the potential value of brief eating-behavior questions as part of health examinations and lifestyle assessment. Current guidelines emphasize weight management, dietary quality, and physical activity as first-line approaches for MASLD [2]. Weight-loss interventions improve biochemical and imaging markers of fatty liver, and larger sustained weight loss can improve steatohepatitis and fibrosis [29,30]. Dietary intervention studies and reviews have further shown that energy restriction and adherence to a Mediterranean-style dietary pattern can improve hepatic fat and metabolic markers [31,32]. The present results suggest that counseling could consider clusters of behaviors—regular breakfast consumption, avoiding meals close to sleep, and a slower eating pace—rather than assigning excessive importance to a single habit. The 2-year landmark analysis further supports this pattern-based interpretation. In that analysis, skipping breakfast and the cumulative unfavorable eating behavior score remained associated with incident MASLD/MetALD, whereas the associations for eating dinner within 2 h before bedtime and fast eating were attenuated. These findings suggest that clinicians should consider the overall clustering of unfavorable eating behaviors rather than targeting each behavior in isolation, while recognizing that skipping breakfast showed the most consistent individual association in the present study. Intervention studies are required, however, before concluding that modifying these behavioral patterns can lower MASLD/MetALD incidence.

This study has several strengths. It included a large number of participants and incident events, used repeated abdominal ultrasonography rather than a single cross-sectional assessment, and evaluated both individual behaviors and a cumulative score. The sensitivity analyses addressed nonproportional hazards, changes in exposure, time-varying covariates, within-participant correlation, early events, and potential effect modification. The consistency of the continuous score across the primary, time-dependent, and landmark analyses, and the sensitivity analysis censoring participants at the first follow-up examination with newly identified exclusion criteria support the stability of the main association.

Several limitations should also be considered. First, the retrospective design and the requirement for repeated health examinations may have introduced selection bias because individuals who returned for follow-up may differ in health awareness, occupation, or lifestyle from those who did not. Second, eating behaviors, sleep-related rest, and alcohol consumption were self-reported. The questionnaire did not quantify total energy intake, macronutrient composition, overall dietary quality, exact meal timing, eating duration, chronotype, shift work, occupational activity, sedentary behavior, sitting time, food insecurity, or socioeconomic status; exposure misclassification and residual confounding are therefore unavoidable. In particular, sedentary occupation and prolonged sitting time may influence total energy expenditure and may also be associated with clustering of unfavorable eating behaviors, such as skipping breakfast and eating dinner close to bedtime. Third, hepatic steatosis was identified by ultrasonography, which is suitable for large health-examination cohorts but is less sensitive for mild steatosis and cannot quantify liver fat or distinguish steatosis from steatohepatitis [33]. Fourth, the true onset of MASLD occurred between examinations, and assigning the event to the examination date introduced interval uncertainty. Fifth, the operational outcome combined conventional MASLD and MetALD after excluding alcohol intake above the prespecified threshold. Although both categories require hepatic steatosis and cardiometabolic dysfunction within the consensus framework [1], this approach may obscure heterogeneity related to moderate alcohol exposure, and self-reported alcohol intake may cause misclassification. Sixth, time-updated adjustment for metabolic variables may have controlled partly for mediators and should not be interpreted as an estimate of the total causal effect. Finally, all participants were Japanese adults attending three clinics in Tokyo, which may limit generalizability to other ethnic groups, healthcare settings, and populations with advanced liver disease.

Taken together, the findings indicate that the cumulative eating behavior score was associated with incident MASLD/MetALD, but the evidence was not equally strong for all three component behaviors. Skipping breakfast showed the most consistent association, whereas eating dinner within 2 h before bedtime and fast eating showed smaller and less consistent associations across sensitivity analyses. The persistence of the cumulative score suggests that the overall behavioral pattern may be more informative than any single component, although this should not be interpreted as evidence that all three behaviors contribute equally. Prospective studies incorporating objective meal-timing data, detailed dietary assessment, quantitative liver-fat imaging, and methods that appropriately address time-varying confounding are warranted. Randomized or pragmatic interventions are needed to determine whether modifying these behaviors can lower incident MASLD/MetALD independently of weight loss.

## 5. Conclusions

The accumulation of unfavorable eating behaviors was associated with a progressively higher hazard of incident MASLD/MetALD. Assessing multiple eating behaviors simultaneously, rather than a single habit, may provide additional information for lifestyle assessment and risk stratification, particularly in non-obese individuals.

## Figures and Tables

**Table 1:** Baseline characteristics according to the number of unfavorable eating behaviors.

Characteristics	Overall *N* = 29,889	0 Behaviors *N* = 10,441	1 Behavior *N* = 11,343	2 Behaviors *N* = 6392	3 Behaviors *N* = 1713	*p*-Value ^1^
Age, years	45.3 ± 9.5	46.9 ± 10.0	45.5 ± 9.5	43.3 ± 8.4	42.0 ± 7.7	<0.001
Male sex	13,398 (44.8%)	3477 (33.3%)	5142 (45.3%)	3675 (57.5%)	1104 (64.4%)	<0.001
Current smoking	4490 (14.6%)	841 (8.1%)	1514 (13.3%)	1525 (23.9%)	610 (35.6%)	<0.001
Alcohol consumption, g/week	30.0 [0.0, 70.0]	30.0 [0.0, 60.0]	30.0 [0.0, 70.0]	30.0 [0.0, 100.0]	60.0 [0.0, 210.0]	<0.001
Regular exercise for ≥30 min	6828 (22.8%)	2402 (23.0%)	2665 (23.5%)	1412 (22.1%)	349 (20.4%)	0.012
Physical activity for ≥1 h/day	13,475 (45.1%)	4822 (46.2%)	5125 (45.2%)	2773 (43.4%)	755 (44.1%)	0.004
Adequate rest from sleep	18,878 (62.9%)	7016 (67.2%)	7143 (63.0%)	3699 (57.9%)	930 (54.3%)	<0.001
History of hypertension	1586 (5.3%)	568 (5.4%)	669 (5.9%)	285 (4.5%)	64 (3.7%)	<0.001
History of diabetes mellitus	268 (0.9%)	85 (0.8%)	126 (1.1%)	52 (0.8%)	5 (0.3%)	0.003
History of dyslipidemia	1304 (4.4%)	537 (5.1%)	521 (4.6%)	191 (3.0%)	55 (3.2%)	<0.001
Body mass index, kg/m^2^	21.4 ± 2.6	21.0 ± 2.5	21.5 ± 2.6	21.8 ± 2.6	22.1 ± 2.7	<0.001
Waist circumference, cm	78.1 ± 7.9	76.8 ± 7.8	78.4 ± 7.9	79.3 ± 7.8	80.4 ± 7.9	<0.001
Systolic blood pressure, mmHg	112.5 ± 15.3	112.2 ± 15.9	112.6 ± 15.3	112.8 ± 14.8	113.1 ± 14.6	0.018
Fasting plasma glucose, mg/dL	86.0 ± 10.4	85.8 ± 10.3	86.1 ± 10.7	86.3 ± 10.2	85.9 ± 9.5	0.014
Triglycerides, mg/dL	69.0 [52.0, 94.0]	66.0 [51.0, 89.0]	69.0 [52.0, 95.0]	72.0 [55.0, 101.0]	75.0 [56.0, 103.0]	<0.001
HDL cholesterol, mg/dL	68.7 ± 17.0	71.1 ± 17.0	68.6 ± 17.0	66.2 ± 16.6	64.6 ± 16.2	<0.001
Aspartate aminotransferase, U/L	19.0 [16.0, 22.0]	19.0 [16.0, 22.0]	19.0 [16.0, 22.0]	19.0 [16.0, 22.0]	19.0 [16.0, 22.0]	0.322
Alanine aminotransferase, U/L	15.0 [11.0, 20.0]	15.0 [11.0, 19.0]	15.0 [11.0, 20.0]	16.0 [12.0, 21.0]	16.0 [12.0, 22.0]	<0.001
γ-Glutamyl transferase, U/L	19.0 [14.0, 29.0]	18.0 [13.0, 26.0]	19.0 [14.0, 29.0]	20.0 [15.0, 33.0]	22.0 [15.0, 35.0]	<0.001
Uric acid, mg/dL	5.1 ± 1.3	4.8 ± 1.2	5.1 ± 1.3	5.4 ± 1.3	5.5 ± 1.4	<0.001
eGFR, mL/min/1.73 m^2^	79.9 ± 14.1	79.4 ± 14.3	79.8 ± 14.2	80.7 ± 13.7	81.7 ± 13.4	<0.001

Data are presented as Mean ± SD, *n* (%), or Median [Q1, Q3]; ^1^ One-way ANOVA; Chi-squared test; Kruskal–Wallis rank sum test.

**Table 2:** Associations of individual unfavorable eating behaviors with incident MASLD/MetALD.

Eating Behavior	Status	N	Events	Incidence Rate per 1000 Person-Years	Crude HR (95% CI)	Model 1 HR (95% CI)	Model 2 HR (95% CI)	Model 3 HR (95% CI)	Model 4 HR (95% CI)	Mutually Adjusted Model 4 HR (95% CI)
Skipping breakfast	No	22,859	3501	39.4	1.00 (reference)	1.00 (reference)	1.00 (reference)	1.00 (reference)	1.00 (reference)	1.00 (reference)
	Yes	7030	1328	50.6	1.29 (1.21–1.37)	1.24 (1.16–1.33)	1.23 (1.15–1.32)	1.24 (1.16–1.32)	1.22 (1.14–1.30)	1.19 (1.11–1.27)
Eating dinner within 2 h before bedtime	No	19,007	2731	37.6	1.00 (reference)	1.00 (reference)	1.00 (reference)	1.00 (reference)	1.00 (reference)	1.00 (reference)
	Yes	10,882	2098	49.4	1.31 (1.24–1.39)	1.18 (1.11–1.25)	1.23 (1.18–1.28)	1.16 (1.09–1.23)	1.14 (1.08–1.21)	1.11 (1.05–1.18)
Fast eating	No	18,535	2581	36.7	1.00 (reference)	1.00 (reference)	1.00 (reference)	1.00 (reference)	1.00 (reference)	1.00 (reference)
	Yes	11,354	2248	50.1	1.36 (1.29–1.44)	1.26 (1.19–1.34)	1.28 (1.20–1.35)	1.08 (1.01–1.14)	1.08 (1.02–1.14)	1.06 (1.00–1.13)

Incidence rates are expressed per 1000 person-years. HR, hazard ratio; CI, confidence interval. Model 1 was adjusted for age, sex, smoking status, and log-transformed alcohol consumption. Model 2 was additionally adjusted for regular exercise, physical activity, and adequate rest from sleep. Model 3 was additionally adjusted for body mass index. Model 4 was additionally adjusted for fasting plasma glucose, systolic blood pressure, triglycerides, and HDL cholesterol. The mutually adjusted Model 4 included all three eating behaviors simultaneously together with all Model 4 covariates.

**Table 3:** Cox proportional hazards regression analysis of the association between the number of unfavorable eating behaviors and incident MASLD/MetALD.

Analysis	0 Behaviors	1 Behavior	2 Behaviors	3 Behaviors	Per 1-Behavior Increment
Event data					
*N*	10,441	11,343	6392	1713	—
Events	1280	1858	1257	434	—
Incidence rate per 1000 person-years	32.2	42.5	50.4	65.0	—
Baseline Cox proportional hazards models					
Crude HR (95% CI)	1.00 (reference)	1.32 (1.23–1.42)	1.57 (1.45–1.69)	2.02 (1.82–2.26)	1.25 (1.22–1.29); *p* < 0.001
Model 1 HR (95% CI)	1.00 (reference)	1.24 (1.15–1.33)	1.40 (1.29–1.52)	1.74 (1.55–1.95)	1.19 (1.15–1.23); *p* < 0.001
Model 2 HR (95% CI)	1.00 (reference)	1.24 (1.16–1.34)	1.42 (1.31–1.54)	1.75 (1.56–1.97)	1.20 (1.16–1.24); *p* < 0.001
Model 3 HR (95% CI)	1.00 (reference)	1.11 (1.03–1.19)	1.24 (1.14–1.34)	1.47 (1.31–1.64)	1.13 (1.09–1.16); *p* < 0.001
Model 4 HR (95% CI)	1.00 (reference)	1.12 (1.04–1.20)	1.22 (1.13–1.33)	1.44 (1.28–1.61)	1.12 (1.08–1.15); *p* < 0.001
Cox models with time-dependent covariates					
Updated score, baseline covariates HR (95% CI)	1.00 (reference)	1.11 (1.03–1.19)	1.24 (1.15–1.35)	1.53 (1.34–1.74)	1.13 (1.10–1.18); *p* < 0.001
Updated score and updated covariates HR (95% CI)	1.00 (reference)	1.07 (0.99–1.14)	1.14 (1.05–1.24)	1.33 (1.17–1.52)	1.09 (1.05–1.13); *p* < 0.001

Unfavorable eating behaviors comprised skipping breakfast, eating dinner within 2 h before bedtime, and fast eating. Incidence rates are expressed per 1000 person-years. HR, hazard ratio; CI, confidence interval. Model 1 was adjusted for age, sex, smoking status, and log-transformed alcohol consumption. Model 2 was additionally adjusted for regular exercise, physical activity, and adequate rest from sleep. Model 3 was additionally adjusted for body mass index. Model 4 was additionally adjusted for fasting plasma glucose, systolic blood pressure, triglycerides, and HDL cholesterol. In the first model with time-dependent covariates, the eating behavior score was updated at each examination, whereas adjustment covariates were fixed at baseline. In the exploratory updated-covariate model, both the eating behavior score and the available covariates were updated at each examination. Robust variance estimates were clustered by participant. *p* values shown for the per 1-behavior increment correspond to the continuous eating behavior score.

**Table 4:** Landmark sensitivity analyses of the associations of unfavorable eating behaviors with incident MASLD/MetALD.

Exposure	1-Year Landmark *N* = 24,813; Events = 4106		2-Year Landmark *N* = 18,499; Events = 2896	
	Adjusted HR (95% CI)	*p* Value	Adjusted HR (95% CI)	*p* Value
Individual eating behaviors				
Skipping breakfast (yes vs. no)	1.22 (1.13–1.31)	<0.001	1.18 (1.08–1.29)	<0.001
Eating dinner within 2 h before bedtime (yes vs. no)	1.09 (1.02–1.17)	0.008	1.06 (0.98–1.14)	0.180
Fast eating (yes vs. no)	1.06 (1.00–1.13)	0.053	1.05 (0.97–1.13)	0.225
Unfavorable eating behavior score				
0 behaviors	1.00 (reference)	—	1.00 (reference)	—
1 behavior	1.15 (1.06–1.25)	<0.001	1.11 (1.01–1.21)	0.034
2 behaviors	1.24 (1.13–1.35)	<0.001	1.17 (1.05–1.30)	0.004
3 behaviors	1.43 (1.26–1.62)	<0.001	1.31 (1.13–1.53)	<0.001
Per 1-behavior increment	1.12 (1.08–1.16)	<0.001	1.09 (1.04–1.13)	<0.001

Values are hazard ratios (HRs) with 95% confidence intervals (CIs). The 1-year and 2-year landmark analyses included participants who remained free of MASLD/MetALD and under observation beyond the corresponding landmark time; follow-up was recalculated from the landmark time. Models were adjusted for age, sex, smoking status, log-transformed alcohol consumption, regular exercise, physical activity, adequate rest from sleep, body mass index, fasting plasma glucose, systolic blood pressure, triglycerides, and high-density lipoprotein cholesterol. The individual eating behavior models additionally included the other two eating behaviors. Abbreviations: CI, confidence interval; HR, hazard ratio; MASLD, metabolic dysfunction-associated steatotic liver disease; MetALD, metabolic dysfunction- and alcohol-associated liver disease.

**Table 5:** Subgroup analyses of the association between the unfavorable eating behavior score and incident MASLD/MetALD.

Effect Modifier	Subgroup	*N*	Events	Adjusted HR per 1-Behavior Increment (95% CI)	*p* Value	*p* for Interaction
Sex	Female	16,491	1781	1.06 (1.00–1.12)	0.043	0.109
	Male	13,398	3048	1.13 (1.08–1.17)	<0.001	
Body mass index	BMI < 23 kg/m^2^	22,368	2509	1.19 (1.14–1.24)	<0.001	<0.001
	BMI ≥ 23 kg/m^2^	7521	2320	1.08 (1.03–1.13)	0.002	

Hazard ratios represent the association with incident MASLD/MetALD per one-behavior increment in the unfavorable eating behavior score. Sex-stratified models were adjusted for age, smoking status, log-transformed alcohol consumption, regular exercise, physical activity, adequate rest from sleep, body mass index, fasting plasma glucose, systolic blood pressure, triglycerides, and HDL cholesterol. BMI-stratified models were adjusted for age, sex, smoking status, log-transformed alcohol consumption, regular exercise, physical activity, adequate rest from sleep, fasting plasma glucose, systolic blood pressure, triglycerides, and HDL cholesterol. *p*-values for interaction were obtained from likelihood ratio tests comparing models with and without the interaction term between the unfavorable eating behavior score and the corresponding effect modifier. Subgroup analyses were exploratory. HR, hazard ratio; CI, confidence interval; BMI, body mass index; HDL, high-density lipoprotein; MASLD, metabolic dysfunction-associated steatotic liver disease; MetALD, metabolic dysfunction- and alcohol-associated liver disease.

## Data Availability

The data supporting the findings of this study are available from the corresponding author upon reasonable request due to privacy and confidentiality restrictions concerning individual-level health-examination data.

## Supplementary Materials

The following supporting information can be downloaded at https://www.mdpi.com/article/10.3390/nu18182957/s1, Supplementary Table S1. Baseline characteristics of eligible individuals according to follow-up status; Supplementary Table S2. Extended Cox model for individual unfavorable eating behaviors; Supplementary Table S3. Extended Cox model for the unfavorable eating behavior score; Supplementary Table S4. Time-dependent analyses of individual unfavorable eating behaviors and incident MASLD/MetALD; Supplementary Table S5. Sensitivity analysis censoring participants at the first follow-up examination with newly identified exclusion criteria; Supplementary Figure S1. Flow chart of the study population.

## Abbreviations

The following abbreviations are used in this manuscript:

BMI	Body mass index
CI	Confidence interval
FPG	Fasting plasma glucose
HDL-C	High-density lipoprotein cholesterol
HR	Hazard ratio
IQR	Interquartile range
MASLD	Metabolic dysfunction-associated steatotic liver disease
MetALD	Metabolic dysfunction- and alcohol-associated liver disease
NAFLD	Non-alcoholic fatty liver disease
SBP	Systolic blood pressure
SD	Standard deviation
TGs	Triglycerides
