# Supplementary material for: Accumulation and Time-Dependent Changes in Unfavorable Eating Behaviors and the Risk of Incident Metabolic Dysfunction-Associated and Alcohol-Related/Associated Steatotic Liver Disease: A Retrospective Cohort Study
Source: Nutrients. 2026 Sep 9;18(18):2957. doi: 10.3390/nu18182957 (PMC13610941; doi:10.3390/nu18182957)

Supplementary Table S1. Baseline characteristics of eligible individuals according to follow-up status

Characteristic	Category	Overall<br>N = 53,603	No follow-up examination<br>N = 23,714	Included in incidence cohort<br>N = 29,889	P value	Absolute SMD
Participants, n		53,603	23,714	29,889	—	—
**Demographic and lifestyle characteristics**						
Age, years		45.16 (9.72)	44.97 (10.02)	45.32 (9.48)	<0.001	0.04
Sex, n (%)	Female	29,957 (55.9)	13,466 (56.8)	16,491 (55.2)	<0.001	0.03
	Male	23,646 (44.1)	10,248 (43.2)	13,398 (44.8)	—	—
Current smoking, n (%)	No	45,779 (85.4)	20,380 (85.9)	25,399 (85.0)	0.002	0.03
	Yes	7,824 (14.6)	3,334 (14.1)	4,490 (15.0)	—	—
Alcohol consumption, g/week		0.00 [0.00–70.00]	0.00 [0.00–60.00]	30.00 [0.00–70.00]	<0.001	**0.22**
Regular exercise, n (%)	No	40,975 (76.4)	17,914 (75.5)	23,061 (77.2)	<0.001	0.04
	Yes	12,628 (23.6)	5,800 (24.5)	6,828 (22.8)	—	—
Physical activity ≥1 h/day, n (%)	No	28,683 (53.5)	12,269 (51.7)	16,414 (54.9)	<0.001	0.06
	Yes	24,920 (46.5)	11,445 (48.3)	13,475 (45.1)	—	—
Adequate rest from sleep, n (%)	No	19,464 (36.3)	8,363 (35.3)	11,101 (37.1)	<0.001	0.04
	Yes	34,139 (63.7)	15,351 (64.7)	18,788 (62.9)	—	—
Hypertension, n (%)	No	50,875 (94.9)	22,572 (95.2)	28,303 (94.7)	0.011	0.02
	Yes	2,728 (5.1)	1,142 (4.8)	1,586 (5.3)	—	—
Diabetes, n (%)	No	53,093 (99.0)	23,472 (99.0)	29,621 (99.1)	0.155	0.01
	Yes	510 (1.0)	242 (1.0)	268 (0.9)	—	—
Dyslipidemia, n (%)	No	51,262 (95.6)	22,677 (95.6)	28,585 (95.6)	0.971	0.00
	Yes	2,341 (4.4)	1,037 (4.4)	1,304 (4.4)	—	—
**Unfavorable eating behaviors**						
Skipping breakfast, n (%)	No	40,505 (75.6)	17,646 (74.4)	22,859 (76.5)	<0.001	0.05
	Yes	13,098 (24.4)	6,068 (25.6)	7,030 (23.5)	—	—
Eating dinner within 2 h before bedtime, n (%)	No	34,626 (64.6)	15,619 (65.9)	19,007 (63.6)	<0.001	0.05
	Yes	18,977 (35.4)	8,095 (34.1)	10,882 (36.4)	—	—
Fast eating, n (%)	No	33,871 (63.2)	15,336 (64.7)	18,535 (62.0)	<0.001	0.06
	Yes	19,732 (36.8)	8,378 (35.3)	11,354 (38.0)	—	—
Unfavorable eating behavior score, n (%)	0 behaviors	19,139 (35.7)	8,698 (36.7)	10,441 (34.9)	<0.001	0.04
	1 behavior	20,157 (37.6)	8,814 (37.2)	11,343 (38.0)	—	—
	2 behaviors	11,271 (21.0)	4,879 (20.6)	6,392 (21.4)	—	—
	3 behaviors	3,036 (5.7)	1,323 (5.6)	1,713 (5.7)	—	—
**Anthropometric and laboratory measurements**						
BMI, kg/m²		21.44 (2.67)	21.45 (2.70)	21.42 (2.64)	0.156	0.01
Waist circumference, cm		78.11 (7.94)	78.12 (7.99)	78.10 (7.90)	0.781	0.00
SBP, mmHg		112.96 (15.52)	113.49 (15.72)	112.53 (15.34)	<0.001	0.06
FPG, mg/dL		86.26 (10.64)	86.55 (11.00)	86.03 (10.35)	<0.001	0.05
TG, mg/dL		69.00 [52.00–95.00]	70.00 [52.00–96.00]	69.00 [52.00–94.00]	0.128	0.02
HDL-C, mg/dL		69.03 (17.21)	69.40 (17.42)	68.74 (17.04)	<0.001	0.04
AST, U/L		19.00 [16.00–22.00]	19.00 [16.00–22.00]	19.00 [16.00–22.00]	0.033	0.03
ALT, U/L		15.00 [12.00–20.00]	15.00 [12.00–21.00]	15.00 [11.00–20.00]	<0.001	0.03

Characteristic	Category	Overall N = 53,603	No follow-up examination N = 23,714	Included in incidence cohort N = 29,889	P value	Absolute SMD
GGT, U/L		19.00 [14.00–29.00]	19.00 [13.00–28.00]	19.00 [14.00–29.00]	<0.001	0.02
Uric acid, mg/dL		5.06 (1.31)	5.03 (1.31)	5.08 (1.31)	<0.001	0.04
eGFR, mL/min/1.73 m²		79.96 (14.22)	80.00 (14.38)	79.93 (14.09)	0.535	0.01

Values are mean (SD), median [IQR], or n (%), unless otherwise indicated. P values were calculated using Welch's t test, the Kruskal–Wallis test, or the chi-square test, as appropriate. Because of the large sample size, between-group differences were interpreted primarily using the absolute standardized mean difference (SMD); an absolute SMD ≥0.10 was considered potentially meaningful. Participants included in the incidence cohort underwent at least one health examination after baseline. BMI, body mass index; SBP, systolic blood pressure; FPG, fasting plasma glucose; TG, triglycerides; HDL-C, high-density lipoprotein cholesterol; AST, aspartate aminotransferase; ALT, alanine aminotransferase; GGT, γ-glutamyl transferase; eGFR, estimated glomerular filtration rate.

Supplementary Table S2. Extended Cox model for individual unfavorable eating behaviors

Exposure	Comparison	Adjusted HR (95% CI)	P value
Skipping breakfast	Yes vs. no	1.19 (1.11–1.27)	<0.001
Eating dinner within 2 h before bedtime	Yes vs. no	1.11 (1.05–1.18)	<0.001
Fast eating	Yes vs. no	1.06 (1.00–1.13)	0.034

Supplementary Table S3. Extended Cox model for the unfavorable eating behavior score

Exposure	Comparison	Adjusted HR (95% CI)
0 behaviors	Reference	1.00 (reference)
1 behavior	vs. 0 behaviors	1.12 (1.04–1.20)
2 behaviors	vs. 0 behaviors	1.22 (1.12–1.32)
3 behaviors	vs. 0 behaviors	1.43 (1.28–1.61)
Per 1-behavior increment	Continuous score	1.12 (1.08–1.15)

The extended Cox models stratified the baseline hazard by sex and included time-varying coefficients for age, fasting plasma glucose, systolic blood pressure, and triglycerides through interactions with log(t + 0.01). The individual-behavior model included all three eating behaviors simultaneously and the full set of Model 4 covariates. HR, hazard ratio; CI, confidence interval; FPG, fasting plasma glucose; SBP, systolic blood pressure; TG, triglycerides; MASLD, metabolic dysfunction-associated steatotic liver disease; MetALD, metabolic dysfunction- and alcohol-associated liver disease.

Supplementary Table S4. Time-dependent analyses of individual unfavorable eating behaviors and incident MASLD/MetALD

Exposure	Time-dependent model	Adjusted HR (95% CI)	P value
Skipping breakfast	Updated eating behaviors; baseline covariates	1.22 (1.13–1.30)	<0.001
	Updated eating behaviors and updated covariates	1.15 (1.07–1.23)	<0.001
Eating dinner within 2 h before bedtime	Updated eating behaviors; baseline covariates	1.13 (1.06–1.20)	<0.001
	Updated eating behaviors and updated covariates	1.07 (1.00–1.14)	0.040
Fast eating	Updated eating behaviors; baseline covariates	1.08 (1.01–1.14)	0.015
	Updated eating behaviors and updated covariates	1.05 (0.99–1.12)	0.077

The model with updated eating behaviors and baseline covariates included 29,889 participants who contributed 82,479 observation intervals; 4,829 incident MASLD/MetALD events were observed. In the fully updated model, 52 observation intervals with missing updated covariate values were excluded; this model included 82,427 observation intervals and 4,827 events. In the first model, the three eating behaviors were updated at each examination and entered simultaneously, whereas adjustment covariates were fixed at baseline. In the exploratory model, the three eating behaviors and the available demographic, lifestyle, anthropometric, and metabolic covariates were updated at each examination; sex remained fixed. Both models used robust variance estimates clustered by participant.

Supplementary Table S5. Sensitivity analysis censoring participants at the first follow-up examination with newly identified exclusion criteria

Exposure	Comparison	Adjusted HR (95% CI)	P value
Skipping breakfast	Yes vs. no	1.19 (1.11–1.28)	<0.001
Eating dinner within 2 h before bedtime	Yes vs. no	1.10 (1.04–1.17)	0.002
Fast eating	Yes vs. no	1.06 (1.00–1.12)	0.067
**Unfavorable eating behavior score**	0 behaviors	1.00 (reference)	—
	1 behavior vs. 0 behaviors	1.11 (1.03–1.19)	0.007
	2 behaviors vs. 0 behaviors	1.20 (1.11–1.31)	<0.001
	3 behaviors vs. 0 behaviors	1.41 (1.26–1.59)	<0.001

Exposure	Comparison	Adjusted HR (95% CI)	P value
	Per 1-behavior increment	1.11 (1.07–1.15)	<0.001

Participants were censored at the first follow-up examination at which excessive alcohol consumption, malignancy, or thyroid disease was newly identified. This sensitivity analysis included 29,889 participants, 4,627 incident MASLD/MetALD events, and 112,726 person-years of follow-up; 969 participants were censored because of newly identified exclusion criteria during follow-up. The censoring criteria were excessive alcohol consumption in 373 participants, malignancy in 578 participants, and thyroid disease in 24 participants. Because some participants met more than one criterion at the censoring examination, the reason-specific counts are not mutually exclusive. Models were adjusted using the same covariates as Model 4. The individual eating behavior model included all three eating behaviors simultaneously. HR, hazard ratio; CI, confidence interval; MASLD, metabolic dysfunction-associated steatotic liver disease; MetALD, metabolic dysfunction- and alcohol-associated liver disease.

Supplementary Figure S1. Flow chart of the study population.

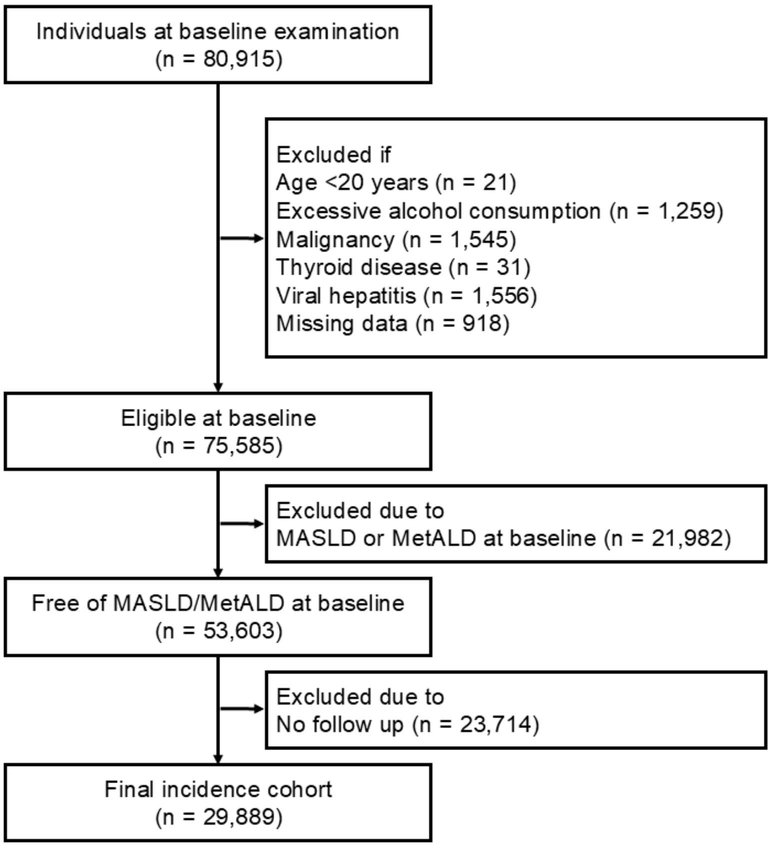

Supplement: Supplementary file 1 [file nutrients-18-02957-s001.zip › nutrients-4546666-supplementary.pdf]
